# Cerebral Vasculopathy and Spinal Arachnoiditis: Two Rare Complications of Ventriculitis Post Subarachnoid Hemorrhage

**DOI:** 10.7759/cureus.12241

**Published:** 2020-12-23

**Authors:** Parneet Grewal, Julianne P Hall, Miral Jhaveri, Rima M Dafer

**Affiliations:** 1 Neurology, Medical University of South Carolina, Charleston, USA; 2 Neurology, Rush University Medical Center, Chicago, USA; 3 Radiology, Rush University Medical Center, Chicago, USA

**Keywords:** froin syndrome, vasculopathy, subarachnoid hemorrhage, ventriculitis, spinal arachnoiditis

## Abstract

This report describes a case of subarachnoid hemorrhage complicated by ventriculitis and subsequent delayed cerebral vasospasm, severe chronic spinal arachnoiditis, and Froin syndrome. A 60-year-old woman presented with diffuse aneurysmal subarachnoid hemorrhage and underwent successful coil embolization of ruptured left anterior cerebral artery aneurysm. Her course was complicated by bacterial ventriculitis and acute hydrocephalus necessitating ventriculoperitoneal shunt placement. She returned ten weeks later with recurrent headaches; CT angiography showed diffuse cerebral vasospasm. Spine magnetic resonance imaging ordered due to concern for mass or other obstruction of the cerebrospinal fluid obstruction based on lumbar puncture results showed leptomeningeal enhancement with loculated cerebrospinal fluid collections along the spinal canal concerning for spinal arachnoiditis and septal adhesions. Lumbar puncture was consistent with Froin syndrome. She was treated with calcium-channel blockers. Follow up imaging showed resolution of vasospasm, but progression of the arachnoiditis. No surgical intervention was pursued as the patient had no symptoms concerning myelopathy. Aneurysmal subarachnoid hemorrhage and ventriculitis may lead to delayed reversible vasculopathy as well as arachnoiditis, with “dry tap” and Froin-like syndrome picture. Workup should be initiated in patients who develop persistent headaches or myelopathic changes to investigate these possibilities.

## Introduction

Thunderclap headache (TCH) is defined as a sudden severe headache, which reaches maximum intensity within seconds to less than one minute and is described as the worst headache of someone’s life [1, 2]. Aneurysmal SAH (aSAH) is a common cause of TCH and carries high morbidity and mortality, with 10-20% of patients dying before reaching the hospital [3]. Various vascular, infectious, and inflammatory complications can result from aSAH. Here we describe a patient who presented with TCH due to aSAH leading to complications of ventriculitis and subsequent delayed cerebral vasospasm, severe chronic spinal arachnoiditis, and Froin syndrome.

## Case presentation

**Figure 1:**
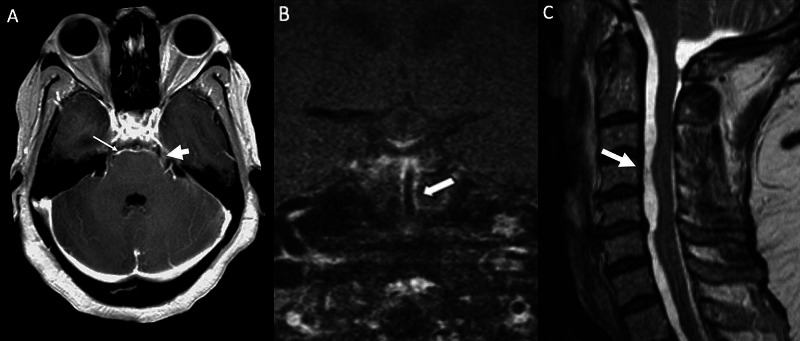
MRI brain and cervical spine Post-contrast axial T1 MR brain (Figure 2A) demonstrates leptomeningeal enhancement along the surface of the pons (arrow) and the cisternal segments of the trigeminal nerves (block arrow). Post-contrast coronal T1 MR (Figure 2B) shows wall enhancement along the basilar artery. Sagittal T2 MR of the spine (Figure 2C) shows loculated CSF collections along the ventral spinal canal in the cervical region associated with cord deformity.

A 60-year-old woman presented with TCH, lightheadedness, and neck stiffness while having a bowel movement. The initial neurological examination was normal with a Glasgow coma score of 15 and Hunt & Hess Classification Grade of one. Over the next 24 hours, she became drowsy, although arousable to voice with no focal deficits. Head computed tomography (CT) scan showed diffuse subarachnoid hemorrhage (SAH) in the basal cisterns, sylvian fissure, and frontoparietal sulci, with intraventricular hemorrhage and mild hydrocephalus, with modified Fisher scale grade of four. CT angiography (CTA) showed a saccular 5 x 2 mm left anterior cerebral artery (ACA) aneurysm. She became obtunded and was subsequently intubated due to respiratory compromise. An external ventricular drain (EVD) was placed due to acute hydrocephalus. She underwent coil embolization of the ACA aneurysm and was successfully extubated, with good initial recovery without any neurological deficit. She subsequently developed Enterobacter aerogenes ventriculitis (protein:165 mg/dL, glucose:9 mg/dL, RBC:12,000, WBC: 6684). Magnetic resonance imaging (MRI) showed diffuse leptomeningeal enhancement (Figure 1) and dependent exudates, necessitating EVD removal. She completed a four-week course of antibiotic therapy with cefepime and had a ventriculoperitoneal shunt placed prior to being discharged home.

**Figure 2:**
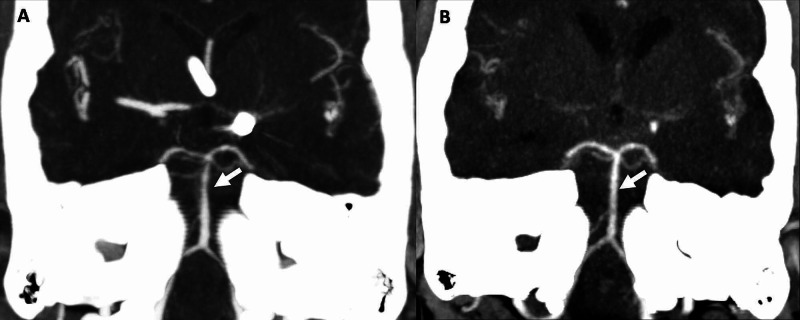
CT angiogram head Coronal MIP CT angiogram image (Figure 2A) shows diffuse narrowing of the distal basilar artery (arrow). Follow up CT angiogram coronal MIP image (Figure 2B) demonstrates resolution of the basilar vasospasm (arrow).

At 10 weeks (day 70) after her initial SAH, she returned to the hospital with a new-onset mild to moderate unilateral pressure-type headache. General and neurological examinations were unremarkable. Head CT was normal but CTA showed diffuse narrowing and irregularities of the proximal intracranial vessels (Figure 2A). X-ray shunt series was unrevealing and there was interval resolution of the previously noted leptomeningeal enhancement on gadolinium-enhanced brain MRI. MR angiogram with vessel wall imaging showed vasospasm with subtle circumferential wall enhancement (Figure 1B). Lumbar puncture resulted in a “dry tap”, i.e. very minimal CSF flow despite accurate needle placement within the subarachnoid space. Analysis of the CSF showed a sterile profile significant for xanthochromia, “thick viscous” CSF, and very elevated CSF protein level (color: xanthochromia, protein 1,428 mg/dL, glucose 36 mg/dL, RBC 238, WBC 12, and negative cultures). In light of the lumbar puncture results, there was a concern for possible mass or other obstruction of CSF within the spinal canal. Therefore, cervical spine MRI was ordered and showed loculated CSF collections along the ventral spinal canal in the cervical and thoracic region concerning spinal arachnoiditis and septal adhesions (Figure 1C) which were attributed to the CNS infection.

She was treated with verapamil due to concern for vasospasm. A follow-up head CTA four weeks later (day 96) showed resolution of the previously reported intracranial arterial narrowing and wall irregularities (Figure 2B). Due to continuing intermittent headaches, further imaging was obtained, with non-revealing head CT and CSF analysis. The surveillance catheter angiogram showed a secured aneurysmal coil and normal intracranial arteries caliber and no vasospasm. There was evidence of worsening arachnoiditis on the MRI spine, with numerous subarachnoid adhesions and loculations throughout the spinal canal associated with multifocal cord deformity as well as clumping of the cauda equina nerve roots. A repeat spine MRI six weeks later was concerning for the ongoing progression of spinal arachnoiditis with multifocal cord deformities as well as clumping of the cauda equina nerve roots. As she remained asymptomatic aside from the headache, with no myelopathic signs, neurosurgery did not recommend any intervention for the arachnoiditis.

## Discussion

aSAH accounts for two to seven percent of all strokes and is a common cause of TCH [3]. Cerebral vasospasm, one of the most common causes of morbidity in aSAH is thought to be related to extravasation of arterial blood, with oxyhemoglobin implicated as one of the main triggers [4]. Cerebral vasospasm typically occurs between 72 hours up to three to four weeks post-ictus [5]. To our knowledge, this unique presentation of delayed vasospasm 70 days after aSAH has not been reported.

Since the patient’s course was complicated by hydrocephalus requiring EVD placement, and subsequent ventriculitis, we suspect the inflammatory changes from the Enterobacter ventriculitis may have triggered the delayed vasospasm. Cerebral vasculopathy, both vasospasm and vasculitis, is not uncommon after central nervous system infections [6]. The pathogen can bind and infect the endothelium or may trigger an immunological or toxic response that can affect the vasculature [6]. Such vascular complications typically occur early within days following initiation of antibiotic therapy. Delayed cerebral vasculitis or vasculopathy is reported as being a rare occurrence and hypothesized to be an effect of immune-mediated mechanisms rather than angio-invasive or toxic effects of the microorganism [7]. Schut et al. described six patients with pneumococcal meningitis who deteriorated 7-19 days after initial infection and were found to have multiple infarctions due to delayed cerebral vasculopathy and cerebral thrombosis [7]. In all of the patients, CSF was sterile. Several other reports showed delayed vasospasm caused by infections with relation to S. miller, S. pneumoniae and S. aureus microorganisms, with poor outcome and with one patient developing arteriopathy consistent with moyamoya-like syndrome [7]. However, the reversibility of the vasculopathy as seen on follow up head CTA and cerebral angiogram in our patient has not been previously described. In all these cases, treatment of delayed vasculopathy consisted of treatment of the underlying infection with antibiotics with or without steroids [7]. Reversible cerebral vasoconstriction syndrome (RCVS) is another cause of TCH and cerebral vasospasm or arterial narrowing that should be considered in such clinical presentations. TCH is typically recurrent in RCVS, and, may often be associated with convexity SAH [1, 2]. The lack of recurrent TCH, the localized vessel wall enhancement, and the abnormal CSF composition are atypical for RCVS, and are more consistent with CNS inflammatory process [2].

Another important finding to mention for this case is highly elevated protein concentration on CSF analysis performed on day 70. While aSAH and inflammation can raise CSF protein concentration, findings of dry tap, xanthochromia, and abnormal CSF with high protein content (> 1000 mg/dL) raised concerns of Froin syndrome-like pattern. This triad of xanthochromia, elevated protein, and hyper-coagulated CSF was first described by Georges Froin in 1910 in a patient with known spinal cord tumor [8]. The development of this syndrome is aided by venous congestion below the level of the compression [8]. Froin syndrome has been described in spinal cord tumors, infections, and degenerative processes. In our patient, despite the resolution of meningeal enhancement and sterile CSF, Froin syndrome-like phenomenon developed, and was suspected to be due to septi formation associated with spinal arachnoiditis in the spinal canal following the aSAH. Spinal arachnoiditis is a rare phenomenon first described by Horsley in 1909 that is most commonly seen following multiple spinal procedures, disc herniation, contrast myelography, central nervous system (CNS) infections-particularly tuberculosis or fungal infections in immunocompromised individuals, and spinal anesthesia and more common in females compared with males [9, 10]. In this patient, possible associated factors could have been her history of ventriculitis, aSAH, and/or prior VP shunt placement. Spinal arachnoiditis has been previously described in SAH due to posterior circulation aneurysms; in our case, we suspect that Spinal arachnoiditis was likely a complication of ventriculitis rather than the SAH [11]. The intermittent episodes of headaches could partially be explained by the vasculopathy and partially by the spinal arachnoiditis with headache mechanism resembling idiopathic intracranial hypertension. 

## Conclusions

In conclusion, transient delayed cerebral vasculopathy mimicking RCVS should be considered in the differential diagnosis of patients who develop new neurological symptoms after central nervous infections. Additionally, aSAH with or without CNS infection may lead to spinal arachnoiditis, with “dry tap” and Froin-like syndrome picture. Thus, we recommend further imaging with angiography (CT or MRI) as well as MRI spine imaging in patients with persistent headaches and those who develop myelopathic changes to exclude delayed vasospasm and spinal arachnoiditis respectively.
